# Directional Growth of cm-Long PLGA Nanofibers by a Simple and Fast Wet-Processing Method

**DOI:** 10.3390/ma15020687

**Published:** 2022-01-17

**Authors:** Erik Betz-Güttner, Martina Righi, Silvestro Micera, Alessandro Fraleoni-Morgera

**Affiliations:** 1 Department of Physics, University of Trieste, Via A. Valerio, 2, 34127 Trieste, Italy; ERIK.BETZGUTTNER@phd.units.it; 2 Sant’Anna School of Advanced Studies, The Biorobotics Institute, Viale R. Piaggio, 34, 56025 Pontedera (Pisa), Italy; martina_righi@fas.harvard.edu (M.R.); silvestro.micera@santannapisa.it (S.M.); 3 Department of Engineering and Architecture, University of Trieste, Via A. Valerio, 6/1, 34127 Trieste, Italy; 4 Department of Engineering and Geology, University of Chieti-Pescara, Viale Pindaro 42, 65127 Pescara, Italy

**Keywords:** nanofibers, PLGA, self-assembly, ASB-SANS, directional nanofiber growth, scaffold

## Abstract

The development of aligned nanofibers as useful scaffolds for tissue engineering is an actively sought-for research objective. Here, we propose a novel improvement of an existing self-assembly-based nanofabrication technique (ASB-SANS). This improvement, which we termed Directional ASB-SANS, allows one to produce cm^2^-large domains of highly aligned poly(lactic-co-glycolic acid) (PLGA) nanofibers in a rapid, inexpensive, and easy way. The so-grown aligned PLGA nanofibers exhibited remarkable adhesion to different substrates (glass, polyimide, and Si/SiOx), even when immersed in PBS solution and kept at physiological temperature (37 °C) for up to two weeks. Finally, the Directional ASB-SANS technique allowed us to grow PLGA fibers also on highly heterogeneous substrates such as polyimide-based, gold-coated flexible electrodes. These results suggest the viability of Directional ASB-SANS method for realizing biocompatible/bioresorbable, nanostructured coatings, potentially suitable for neural interface systems.

## 1. Introduction

Numerous processes have been developed for producing nanofibers, such as electrospinning [1,2], phase separation [3,4], template synthesis [5,6], and wire drawing [7]. Except for electrospinning, all these methods are characterized by a limited ability to generate nanofibers at a large scale or by inherent technical limitations of the process. For example, phase separation allows one to obtain nanostructured foams through a passage in solvents, followed by gelation and extraction, but the process is time-consuming and expensive. Furthermore, these nanostructured foams are characterized by nanofibers with highly inhomogeneous diameter distributions, and overall, the process is slow. The template synthesis relies on the use of a nanoporous matrix to be used as a mold, which delivers nanofibers limited in length by the size of the used matrix, hence rarely exceeding a few microns. Wire drawing methods are limited by the characteristics of the chosen material, which must be viscoelastic and able to withstand the stresses it undergoes during the process without breaking up or developing inhomogeneous diameters.

Another interesting nanofibers fabrication technique is electrospinning, which allows one to obtain indefinitely long single nanofibers in an uninterrupted way from various polymers, potentially on an industrial scale. With a proper instrumental setup, electrospinning can also deliver aligned fibers over considerably large surfaces, up to tens of square centimeters [3]. However, the equipment for carrying out the process is cumbersome and expensive, requiring operations in a dedicated environment, with potential safety hazards linked to the high voltage used in the process.

Self-assembly-based methods have the potential to solve these problems. In fact, self-assembly is the worldwide most diffused nanofabrication process, as it has been chosen by nature for producing living organisms of any size and type, as well as many mineral objects characterized by surprisingly ordered nanostructures [8,9,10]. However, the ability of humans to mimic these natural processes for artificially producing nanofibers has been mainly limited to peptide-like molecules [11,12].

Recently, we proposed a novel self-assembling technique named ASB-SANS (Auxiliary Solvent-Based Sublimation-Aided Nanostructuring), and we showed it to be effective in delivering large-area, hierarchically organized nanofibers, and aligned nanodots [13,14]. The process is based on the use of a ternary solution, consisting of an Auxiliary Solvent (AS), a Sublimating Substance (SS), and a Target Material (TM; the polymer/nano-object to be nanostructured). The TM is chosen so to be well soluble both in the AS and in the SS. Once the solution is deposited on the substrate, the AS evaporation induces the crystallization of the SS/TM solution, which forms a solid crystalline film onto the substrate. Subsequently, the slow sublimation of the crystalline SS forces the migration of the embedded TM within the film, until it reaches the SS crystallites borders, where it precipitates forming the desired nanostructures (nanofibers or aligned nanodots, depending on the ternary solution composition [14]).

Since the whole process is carried out using small amounts of TM (usually between 0.1% and 5% in weight with respect to the SS), after the evaporation of the AS and the consequent solidification the SS maintains its crystalline structure, and the resulting SS crystallites play the role of templates for the TM dissolved in them, leading to well-ordered nanostructures. For higher TM concentrations, the crystalline structure of the SS is disrupted, leading to the formation of disordered TM nanostructures. However, even with appropriately low TM concentrations, the SS crystallizes in randomly oriented polycrystalline domains (possibly very large ones, up to 1 cm-sided [13,14], but usually in the range of tens-hundreds of microns), which arrange themselves as islands, with the associated grain boundaries. Therefore, the so-obtained TM nanostructures grow with appreciable order within the single crystalline domain, but with different alignment orientations at the macro (multidomain) scale. A control over the ratio of the three solution components allows one to obtain different topological arrangements and types of nanostructures (e.g., from ordered arrays of nanofibers to ordered nanodots), with variable degrees of hierarchical development [14]. The ASB-SANS-generated fibers have been demonstrated to be more crystalline than similar fibers obtained using standard methods [15].

The process is extremely inexpensive (relying on simple liquid deposition procedures) and fast (the nanostructures can be grown within half an hour from the deposition of the ternary solution) and can be carried out with common glassware. Moreover, the technique is very versatile in terms of materials, allowing one to produce nanostructures out of different polymers, such as poly(3-hexylthiophene-2,5-diyl) (P3HT), a semiconducting polymer [15,16,17,18], poly(L-lactic acid) (PLLA), a biocompatible polymer [19], poly(methyl methacrylate) (PMMA), a well-known thermoplastic polymer [13,14], and even nano-objects such as carbon nanotubes [13].

Due to this versatility, ASB-SANS-generated nanostructures can be used for a wide range of applications, among which gaseous acetone sensors [15,16,17,18], scaffolds for cell growth and contact guidance studies [19], and self-assembled photolithographic masks for generating silicon monolithic nanopillars with no need for electron beam lithography [14].

Despite this applicative potential, ASB-SANS still suffers from the aforementioned lack of control over the uniform and long-range orientation of the generated nanostructures, which is a highly desirable property in view of growing biological tissues sensitive to the topographic characteristics of the underlying substrate [20].

To fill this gap, we present here a variation of the ASB-SANS technique able to generate uniformly oriented and aligned nanofibers out of PLGA, achieving alignment length exceeding 5 cm and overall aligned nanofibers domains larger than 10 cm^2^. These results are obtained using a seed SS crystal as a multiepitaxial growth template for the solidifying TM-SS layer. In more detail, the seed crystal is put in physical contact with the metastable TM-SS liquid mixture immediately after the AS evaporation, so as to impart a preferential growth direction to the growing polycrystalline layer. Albeit carried out using manual procedures, the here-presented process can be easily automated, opening up interesting opportunities toward the controlled generation of highly aligned and macroscopically developed nanofibers in an inexpensive, fast, and versatile fashion.

## 2. Materials and Methods

### 2.1. Materials

Poly(lactic-co-glycolic Acid) (PLGA) (50:50, MW 45000, Sigma-Aldrich, St. Louis, MO, USA), chloroform (CHCl_3_) (≥99%+), para-dichlorobenzene (PDCB) (≥99%), (−)-menthol (≥99%) were purchased from Sigma-Aldrich (St. Louis, MO, USA) and used without further purification. PLGA was used as Target Material (TM), Chloroform as Auxiliary Solvent (AS), and PDCB as Sublimating Substance (SS).

Polyimide resin (PI 2610) was purchased from HD Microsystems (Neu-Isenburg, Germany). Common laboratory glassware/plasticware was used for the solutions preparation.

### 2.2. ASB-SANS Ternary Solutions Preparation

To prepare the ASB-SANS solutions, at first a TM/AS mother solution was prepared, with a 1 mg/1 mL PLGA/CHCl_3_ concentration. In total, 100 μL of mother solution was added to various quantities of Sublimating Substance (SS), as detailed in Table 1, to obtain SS/TM (*w*/*w*) ratios variable from 400 to 50.

### 2.3. Nanofibrous Samples Preparation and Characterization

The chosen solution, once properly mixed, was deposited by careful drop-casting on the selected substrate (vide infra). Preliminary tests were carried out with PDCB as SS but were discarded upon non-satisfactory results, briefly discussed in the Supplementary Material (Table S1 and Figure S1), where the details about the use of PDCB as SS are also given.

For menthol as SS, the standard ASB-SANS method involved the deposition on the substrate of the ternary solution at room temperature (25 °C), and the simple waiting for the AS to evaporate and the SS to sublimate (see refs. [13,14] for the detailed description of the standard ASB-SANS procedure).

For the here-described novel Directional ASB-SANS protocol, the nanofibers growth step was carried out at (29 ± 0.3) °C, on a custom-made thermostated plate with an attached digital thermometer. After the ternary solution deposition and the almost-complete evaporation of the AS (see refs [13,14,15]), a pure menthol single crystal (α phase, length of about 1.5 cm, diameter of about 4 mm) was placed in an arbitrary part of the as-deposited ternary solution, using the crystal as multiepitaxial growth initiator.

Each developed nanostructure sample was examined by optical microscope (Olympus BH-2 microscope equipped with an Olympus camera; Bethlehem, PA, USA)) and, whenever deemed opportune, by Scanning Electron Microscope (SEM; Carl Zeiss Supra 40 Scanning Electron Microscope, Oberkochen, Germany).

In some cases, the generated nanostructures were also characterized with an ATR FT-IR (Shimadzu IRAffinity-1s, Kyoto, Japan), after having been placed for 24 to 48 h in the water pump vacuum, to check for possible residual menthol left in the PLGA nanopatterns.

### 2.4. Substrates

The nanofibers growth was carried out on different substrates, suited for different purposes:

- SiO_x_ wafer chips (purchased from ITME, Warsaw, Poland, with native oxide layer) were washed with acetone and isopropanol, followed by a nitrogen gas drying step; they were used for SEM imaging of the developed nanostructures.
- Round glass slides (d = 8 mm) coated with a polyimide layer (about 400 nm thick), obtained using a thin-film technology protocol adapted from [21]. The slides were differentiated into two different types:
  (a) pristine polyimide surface
  (b) samples treated with oxygen plasma (Gambetti equipment, Binasco (Milan), Italy): 30 s, 150 W, 15 sccm of O_2_, 300 mTorr

The (a)-type surfaces of these samples were used as controls, in order to evaluate the effect of the surface treatment over the substrate wettability against the ASB-SANS solution.

## 3. Results

### 3.1. Optimization of the Formulation of the ASB-SANS Ternary Solution

The outcome of the ASB-SANS procedure is based on a delicate equilibrium between the three solution components: the Auxiliary Solvent (AS), the Target Material (TM), and the Sublimating Substance (SS). The interplay between these three components, and its role in determining the topology and size of the developed nanostructures, were already investigated in detail [14] and are not discussed here.

PLGA is a copolymer formed by glycolic acid and lactic acid as comonomers, in variable proportions. It was chosen as TM due to its useful properties in terms of cell compatibility and tunable (upon modification of the ratio between the comonomers) bioresorbability. In fact, this polymer has repeatedly shown good ability to promote cell adhesion and proliferation. Thanks to these features, it is considered a reference material in the field of scaffold fabrication [22,23,24].

Chloroform was chosen as AS, thanks to its relatively low boiling point (i.e., about 62 °C), which allows the resulting solution to be easily handled and yet produce a solid film in a few minutes from the deposition. Previous infrared studies did not detect any residual presence of chloroform after the ASB-SANS procedure in the formed nanostructures, even when poly(lactic acid) (PLLA), which is structurally very similar to PLGA, was used as TM [19]. This is a fundamental condition for possible uses of the ASB-SANS-generated nanostructures in scaffold fabrication experiments, as chloroform is known to be detrimental for cells viability.

**Figure 1:**
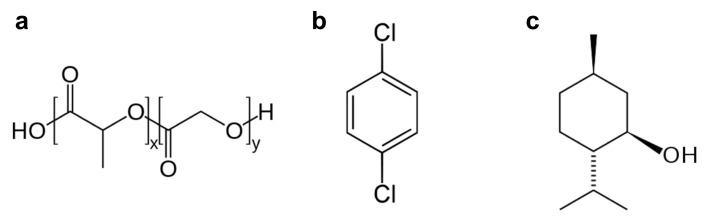
Molecular structures of (**a**) poly(lactic-co-glycolic acid) (PLGA), (**b**) para-dichlorobenzene, and (**c**) (−)-menthol.

We first attempted to obtain a directional growth of the nanofibers using para-dichlorobenzene (PDCB, Figure 1b), which was found to be effective and reliable in previous studies [13,14,15,16,17,18,19]. However, PLGA is not well soluble in PDCB, due to its very hydrophilic structure (Figure 1a).

This resulted in the production of very discontinuous, though remarkably aligned, nanostructures (Figure S1 and related Table S1, Supplementary Material). Nanostructures with similar discontinuous topologies have been demonstrated to be useful to grow retinal progenitor cells [25]. Since our aim was to obtain continuous nanostructures, we replaced PDCB with (−)-menthol, as it resulted in a more suitable SS for our purposes.

Also known as mint camphor, (−)-menthol (or L-menthol, the most common form in nature; from now on, here referred to just as “menthol”) is a chiral cyclic monoterpene alcohol, whose chemical nature allows for enhanced PLGA solubility, due to its alcoholic functional group and its alkylic frame (Figure 1c). It has four crystal polymorphs, of which the most stable one is the α phase (with a crystal habit characterized by short, broad needles) [26]. It is a waxy, crystalline substance, clear or whitish in color, solid at room temperature, with a melting point of 42–45 °C (α phase). It is freely soluble in alcohol, diethyl ether, or chloroform.

Regarding its possible applications in scaffolds for cell growth, menthol is classified as safe by the U.S. Food and Drug Administration (FDA) [27], and several studies revealed a low toxicity in humans [28,29]. Numerous in vitro and in vivo studies have documented biological properties of menthol, such as analgesic, antibacterial, antifungal, anesthetic, and skin-penetration-enhancing effects, as well as immunomodulating actions [30,31,32]. It has also shown the ability to act as inhibitory molecule both on voltage-gated channels that significantly influence functions such as neurotransmission and in gene expression in different cell types [32,33].

Upon the aforementioned properties, we deemed menthol as a suitable SS for our studies, especially thanks to its complete solubility in chloroform, its low melting temperature, its crystalline habit (very favorable for the growth of fibrillar nanopatterns), and its wide and consolidated use in the biomedical field. Due to possible adverse effects on cell viability and growth, after the ASB-SANS process, we dedicated special care to unambiguously rule out the presence of possible menthol residues in the developed nanostructures.

**Figure 2:**
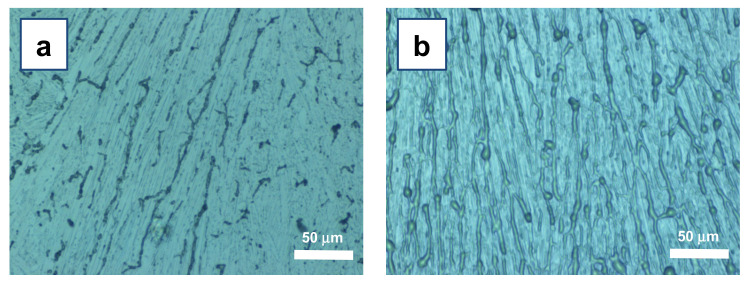
Morphologies of PLGA nanofibers developed using (**a**) low polymer concentrations (sample M400) or (**b**) high polymer concentrations (sample M50). It is possible to appreciate that at low PLGA concentrations, the fibers (dark lines over light blue background) are sparse, often with small lateral size and frequently disrupted, while at high PLGA concentrations, the fibers tend to be overlapped with neighboring ones and to have a large lateral size.

The use of menthol as SS in the ASB-SANS procedure resulted in PLGA fibers with a homogeneous morphology and a width varying from 300 nm to about 4 µm, depending on the initial PLGA concentration: the higher the concentration, the larger the features, in line with previous studies [14,15] (Figure 2). All the samples showed fibrillar patterns with similar topologies and sizes, independently from the type of substrate (Si/SiO_x_ wafer or polyimide-coated glass). FT-IR spectrum analysis did not evidence detectable menthol residues (Figure S2, Supplementary Material), in line with previous investigations [13,14,19].

**Figure 3:**
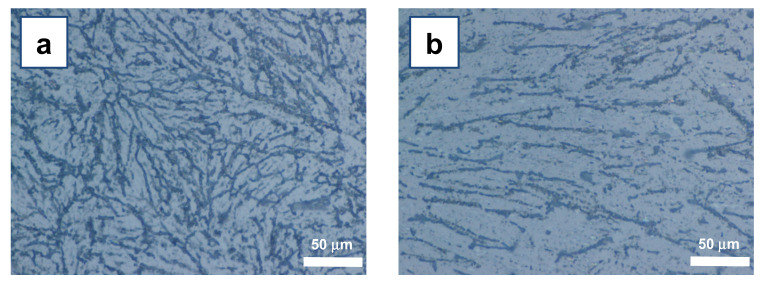
Topology of PLGA nanofibers developed using the M200 solution type, at room temperature upon chloroform evaporation and menthol sublimation, carried out (**a**) in open air, and (**b**) under a closed Petri dish. The imaged area is about 0.85 mm^2^.

Aiming at developing nanofibers suitable for tissue engineering and biomedical applications (e.g., scaffolds and guidance channels for neural cells growth), we identified the M200 as the most suitable composition among the tested ones (see Table 1). The patterns obtained with this system are characterized by continuous nanofibers, rather branched but well distinct from each other, though not extensively aligned (Figure 3a).

### 3.2. Growth of Aligned Nanofibers by Directional ASB-SANS

The obtained PLGA nanofibers, even at the optimized SS/TM ratio of 200, were not uniformly oriented along large surfaces (i.e., surfaces larger than a few tens of μm^2^). Instead, relatively disordered and branched assemblies of nanofibers were obtained.

To obtain the desired alignment, we turned our attention to the directionality of the developed crystallites, which plays a key role in promoting the growth of the nanofibers [13,14]. We hence focused on the mechanisms underlying the solidification phase of the menthol crystal. An accurate literature review showed that, in general, the growth of acicular organic crystals is often complex. Interestingly, a primary threshold energy barrier allows the nucleation and growth of single crystals, but it is not rare that also secondary energy barriers exist for different polymorphs, leading to chaotic and polynuclear/polymorphic crystalline structures [34,35].

In our case, the initial work environment (open air) led to a fast evaporation of chloroform, causing unwanted secondary nucleation centers. In turn, these secondary nucleation centers provided extended seeding, which originated differently oriented and small (a few hundreds of μm^2^ at most) crystal domains. These polycrystalline zones developed into disordered nanofibers domains (Figure 3a).

Our explanation of the observed findings was that secondary nucleation centers were originated by unwanted and uncontrolled local temperature minima, due to spatially uneven chloroform evaporation rates. To overcome this issue, the AS evaporation step of the ASB-SANS process was controlled by keeping the system within a closed system (a closed Petri dish couple), allowing the chloroform vapors to saturate the local atmosphere and considerably slowing down the chloroform evaporation. This approach resulted in an appreciable decrease in menthol nucleation, which in turn delivered an overall more uniform orientation of the fibers, as well as a significant reduction of the branching (Figure 3b). However, these oriented fibers (with domains in the range of a few mm^2^) were disrupted and sparse, likely due to the slow nucleation and precipitation dynamics of PLGA trapped within the menthol crystals [14].

**Figure 4:**
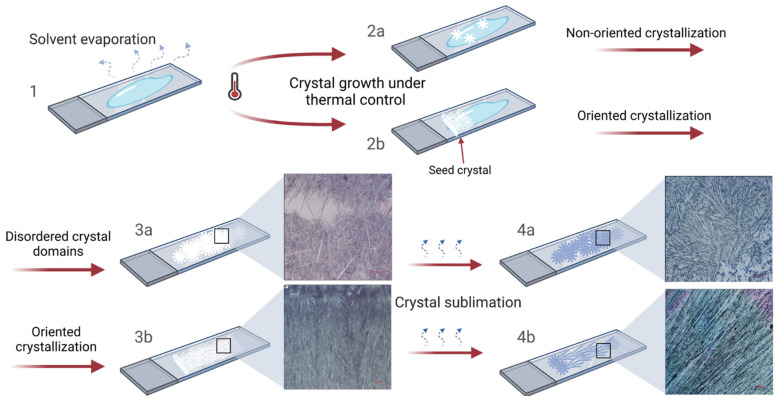
Sketch of the Directional ASB-SANS procedure. At first, the ternary solution is deposited onto the thermostated substrate (step 1). After the deposition, the Auxiliary Solvent slowly evaporates at a controlled temperature, leaving on the substrate a metastable Sublimating Substance/Target Material solid mixture. If the system temperature is properly controlled and a seed crystal of SS is put in physical contact with the metastable mixture, a multiepitaxial directional crystallization of the SS takes place (2b), otherwise a chaotically distributed SS nucleation occurs (2a). The directional crystallization of SS continues until no metastable mixture is left, generating cm-long, aligned SS crystals (3b). If a chaotic nucleation occurs, a disordered polycrystalline SS layer is obtained (3a). The SS crystals sublimation leads hence to the formation of aligned and cm-long (4b) or randomly oriented and relatively short (a few hundreds of microns) (4a) TM nanofibers.

In order to further improve the outcome of the procedure in terms of domain sizes and orientation, we decided to minimize the temperature variations possibly occurring upon uneven heat subtraction operated by the evaporating chloroform. To achieve this result, the initial steps of the ASB-SANS process were carried out at a controlled temperature, from the ternary solution deposition to the starting of the menthol crystallization. In detail, we kept the substrate at around 38 °C, a temperature slightly lower than that of menthol crystallization (about 42–45 °C). This procedure allowed the deposited mixture to remain in liquid phase even after the chloroform was almost completely evaporated (Figure 4), and to keep the menthol in thermal equilibrium at the melt/crystal boundary, achieving the slowest possible crystal growth kinetics.

**Figure 5:**
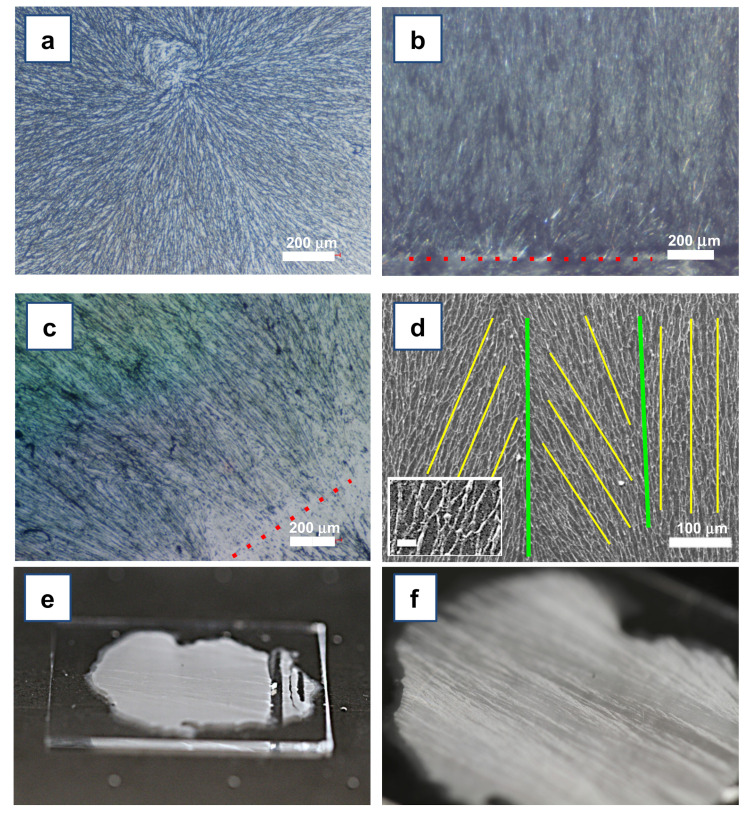
(**a**) Sparse and randomly oriented PLGA nanofibers obtained by standard (nondirectional) ASB-SANS procedure, using menthol as Sublimating Substance. (**b**) Micrograph of aligned PLGA-incorporating menthol crystals obtained using a single crystal of pure menthol as seed for multiepitaxial growth (see Figure S4 for a standard photographic view of the system). The dotted red line at the bottom of the image identifies the edge of the menthol seed crystal. (**c**) Optical micrograph of aligned PLGA nanofibers obtained via the sublimation of menthol from the same system shown in Figure 5b. The red dotted line identifies the main axis of the menthol seed crystal. (**d**) SEM image of the same PLGA nanofibers imaged in Figure 5c by optical microscopy. The green lines identify different domains of orientation of the fibers; the yellow lines highlight the main axis of the domain. In the inset, a magnification of the obtained fibers is shown (scale bar: 10 µm). (**e**,**f**) Optical photographs of menthol/PLGA crystals grown using the Directional ASB-SANS protocol on a glass slide of about 2.5 cm × 5 cm size. (**e**) View of a glass slide coated with aligned crystals grown by the Directional ASB-SANS. The seed crystal was removed to highlight its position during the growth, i.e., in the right part of the slide. (**f**) Close-up of the same sample shown in Figure 5e.

We hence decreased the temperature to 36 °C, and we observed a slow and homogeneous formation of radially oriented menthol/PLGA crystals, with large domains on each considered substrate (Figure S3 and Video SV1, Supplementary Material; Figure 4, steps 1, 2a, 3a). Upon menthol sublimation, the crystalline menthol/PLGA layer evolved in very straight and long PLGA nanofibers, which, however, were found to be sparse and not well aligned. This was attributed to the limited ability of the polymer to diffuse through the large menthol crystals developed upon the slower crystal growth kinetics (Figure 5a).

The so-developed fiber topology (long and aligned but randomly oriented fibers) was only partly in line with our goals. Therefore, we further modified the protocol by placing a seed menthol crystal in direct contact with the metastable solution (Figure S4, Supplementary Material).

The crystal seed, as expected, stimulated the nucleation in the metastable solution, triggering concurrent epitaxial crystallization of menthol at multiple crystal sites (briefly, multiepitaxial crystallization). This phenomenon occurred at a slow rate and generated beautiful, regular, and straight cm-long acicular crystals (Figure 5b). The crystals grew in an overall orthogonal direction with respect to the main axis of the seed crystal, likely due to a higher crystal growth rate along this spatial direction (more experimental work is ongoing to verify this hypothesis, since to the best of the knowledge of the authors a correlation between the crystal habit and the crystal structure of the L-menthol is not yet known). Several crystalline domains grew with a slight tilt (Figure 5b, see to the dotted red line), likely due to some surface irregularities of the seed.

Such an ordered polycrystalline structure obtained with the ASB-SANS procedure provided extremely long and well-ordered PLGA nanofibers (Figure 5c). The SEM image of a typical sample of these nanofibers obtained via the above described Directional ASB-SANS procedure is shown in Figure 5d. The fibers have an approximately round section, with widths ranging from 2–3 to 0.7–0.6 µm (see Figure 5d, inset), where the most typical width is around 1–2 µm. Due to the extended interconnections between the fibers, it was not possible to measure their average length; on the other hand, continuous fibrous patterns for the whole length of the formed menthol crystals, usually in the cm-range, are clearly visible in each obtained sample (see Figure 5, Figure 7a,b, and Figure 8). As expected, since the menthol crystals act as templates for the nanofibers formation [13,14], the orientation of the fibers presented a slight tilt (between a few and 30°) with respect to the perpendicular to the seed crystal.

Overall, the thermal regulation conferred robustness to the growth protocol, allowing reproducibility and granting a reliable control over the directionality of the crystals, and consequently of the nanofibers. With this technique, we were indeed able to obtain menthol crystals with an average lengths >3.5 cm (Figure 5e,f) and, in some cases, up to 6 cm. The nanofibers grew accordingly.

### 3.3. Nanofibers Adhesion and Degradation Tests

**Figure 6:**
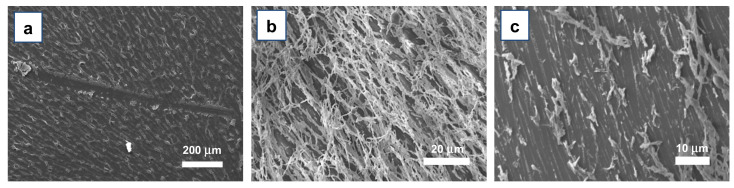
SEM photos of PLGA nanofibers deposited onto Si/SiOx and submitted to different tests. (**a**) fibers scratched with a metal spatula. (**b**,**c**): fibers obtained from a M100 solution before (**b**) and after (**b**) a tape detachment test. In Figure 6c, it is possible to notice that a few nanofibers remained attached to the substrate, though damaged, and that many parts of the fibers remained stuck on the substrate even if their upper part was removed by the tape.

PLGA nanofibers suitable for biological and/or biomedical applications require strong adhesion properties to the targeted substrate, even in the presence of biological fluids. Therefore, we first assessed their adhesion to the substrate by manually scratching the surface of the nanofibers-coated samples with a metallic spatula. Nanofibers grown on Si/SiO_x_ chips (no surface treatment) showed significant substrate adhesion properties: Figure 6a clearly shows that an actual removal of the fibers occurred only in the areas where the metal spatula physically contacted the substrate, while no detectable detachment was found in the surroundings of the scratch. Similar tests were carried out also on other substrates (bare glass, PI-coated glass, and both pristine and UV/oxygen Plasma-treated), with the same results. This good adhesion was likely due to the highly polar nature of PLGA, which allows robust positive interactions with the underlying substrate.

To test more demanding adhesion conditions, we subjected the developed nanofibers to the so-called “tape test”, which consisted of at first pressing on the surface of the sample and then peeling off a strip of common adhesive tape. This test was carried out using the M100 solutions, in order to evaluate the impact on higher density fibers and hence to stress as much as possible their adhesion behavior. Surprisingly, SEM analysis revealed that the tape actually removed only an outer layer of fibers, and that a notable amount of them, though often damaged, remained attached to the substrate (Figure 6b,c), confirming the very good adhesion properties of these ASB-SANS-developed PLGA nanostructures.

Finally, we assessed the ability of the fibers to withstand physiological conditions by immersing supported fibrous samples in phosphate-buffered saline solution (PBS), kept at 37 °C for periods as long as two weeks.

We carried out the first tests of this type on glass/PI substrates, since the PI surface is largely used to develop implantable devices (e.g., invasive neural interfaces) and regenerative electrodes [36,37,38,39]. However, after a few hours of immersion, the PLGA nanofibers fully detached from the pristine PI-coated glass slides.

To counter this problem, we modified the PI surface making it more hydrophilic, so as to increase its interactions with the polar PLGA fibers. In more detail, we treated the PI-coated slides with UV irradiation, followed by oxygen plasma, since it is known that this type of process appreciably increases the amount of hydroxylic groups on PI surfaces [40,41].

We hence grew aligned PLGA nanofibers on the so-treated PI-coated slides, preparing two samples that were immersed in the same PBS solution at 37 °C, and a third one that was used as a reference (not immersed in PBS). The fibers grown on this reference sample were robustly adherent to the oxygen plasma-treated PI, as the tape test resulted in the detachment of the whole PI layer from the underlying glass, rather than in the detachment of the PLGA nanofibers from the PI. After 96 h (4 days) of immersion, the PLGA nanofibers evidenced slight structural modifications under optical microscopy imaging (Figure S5), but the PI layer did not allow for detailed SEM imaging of the sample.

In order to obtain a more accurate morphological characterization of the PLGA nanofibers behavior upon prolonged PBS immersion, we prepared, using the same protocol adopted for the glass/PI substrates, three more samples of Directional ASB-SANS-grown PLGA nanofibers on a substrate more suitable for SEM imaging, i.e., Si/SiO_x_. These samples were tested following the same procedure of immersion in PBS at 37 °C used for the glass/PI substrates.

**Figure 7:**
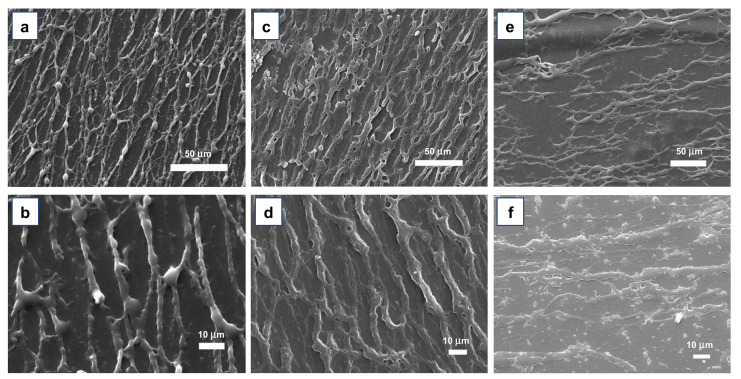
Low and high magnification SEM images of directional ASB-SANS-grown PLGA nanofibers after one hour (**a**,**b**), one week (**c**,**d**), and two weeks (**e**,**f**) of immersion in a PBS bath kept at 37 °C.

The results of these tests are shown in Figure 7. As can be seen, in the reference sample (not immersed in PBS), the fibers show a morphology similar to that of the glass/PI-supported ones (compare Figure 7a to Figure S5a). After one week of PBS treatment, no evidence of detachment or fibers disruption was noticed (Figure 7c,d). In addition, after two weeks of PBS immersion, the fibers still did not show any evident detachment, even though it is possible to observe evident fibers degradation at a high magnification (Figure 7e,f). Moreover, a careful comparison between the reference sample and the immersed ones clearly shows a gradual degradation of the fibers, with progressive flattening, more prominent wrinkled and porous surfaces, roughening and perforations as the time allowed for PBS immersion increases.

These morphological changes are attributed to PLGA swelling consequent to water insertion (absorption) within the fibers and to bulk autocatalytic degradation of the PLGA. In fact, the progressive diffusion of the PBS solution inside the material causes local hydrolysis phenomena, which in turn lead to an accumulation of degradation compounds (mainly glycolic and lactic acid) [42]. These residues, due to the polarity of the PLGA, tend to be adsorbed to the polymer chains, acidifying the local solution and increasing the rate of degradation, in a positive and progressively faster feedback [43]. This degradation process, however, did not seem to affect the adhesion properties of the immersed fibers. In fact, even the sample kept in the PBS solution for two weeks (which showed an enhanced fibers degradation, an evident hydrolysis-induced fibers breakages, large pores, and an overall strong swelling, see Figure 7e,f), kept a satisfactory adherence to the substrate, to the point that the tape detachment tests carried out over the glass/PI-supported fibers resulted in the separation of the PI/nanofibers layer from the glass, rather than the separation of the fibers from the PI. Unfortunately, for periods longer than two weeks, it was not possible to continue the immersion tests, due to the detachment of the whole PI coating layer (including the still adherent PLGA nanofibers) from the glass support, which did not allow for further characterization of the overall fiber degradation behavior.

### 3.4. Growth of PLGA Nanofibers over Heterogeneous Substrates

Neural interfaces are devices able to provide an electrical bridge between the nervous system and an artificial device to restore sensory-motor functions or bioelectronic therapies [44,45,46]. However, the long-term usability of these devices is typically hampered by their nonoptimal biocompatibility or by the mechanical mismatch between the tissue and the device. These problems were addressed by coating the device electrodes with biocompatible materials, such as collagen [47], hydrogels [48], or other organic materials [21,49,50], possibly with thicknesses down to the single-molecule layer [51]. Moreover, it is known that properly nanostructured/nanoporous substrates improve cells’ viability and proliferation [52,53,54].

In this frame, the aligned PLGA fibers produced by directional ASB-SANS can contribute to support and promote neurons growth and axonal sprouting. In fact, aligned fibers are well known to favor the growth and development of neural cells, creating an ideal surface for effective neuronal growth and nerve regeneration [54,55,56,57], and coatings based on bioresorbable polymers can improve the neural tissue growth process [54,55,58,59].

**Figure 8:**
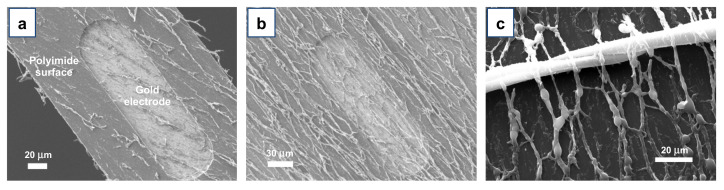
SEM images of Directional ASB-SANS-grown PLGA nanofibers developed over heterogeneous substrates, constituted by a PI and gold, using M400 (**a**) and M200 (**b**) ternary solutions. (**c**) SEM micrograph of a microscopic cotton fiber (large white and almost horizontal fiber) surmounted by numerous PLGA nanofibers, perpendicularly placed on it.

We hence explored the possibility to use Directional ASB-SANS for directly producing aligned PLGA nanofibers onto flexible neural interfaces. In particular, we targeted flexible neural interfaces of the TIME type [60], designed and developed at the Biorobotics Institute of the Sant’Anna University, for in vivo testing in rats [61,62]. Figure 8a,b show the details of one of the conductive areas (active sites) of the device, obtained by etching ~5 µm of the polyimide layer, to expose a small area of the underlying ~250 nm thick gold electrode. Such a heterogeneous surface, both in terms of materials and morphology, does not represent an ideal substrate for the here-described Directional ASB-SANS technique, since it is well known that crystallization is poorly effective in the presence of multiple heterogeneous nucleation sites (in terms of different materials and/or geometrical discontinuities), and ASB-SANS relies on a crystallization step to provide satisfying results.

Despite the apparent unfavorable heterogeneous surface, we decided anyway to carry out the Directional ASB-SANS protocol on the aforementioned neural interfaces. Surprisingly, the PLGA nanofibers did develop very well all over the heterogeneous surface (Figure 8a,b), with no visible discontinuities or changes in fiber alignment direction and morphology. This was verified even using different ternary solution formulations, i.e., the M200 and M400. Moreover, we observed how the presence of external impurities such as macroscopic textile fibers did not affect the growth and directionality of the fibers (Figure 8c), demonstrating a good robustness of the Directional ASB-SANS technique and its suitability for a broad set of substrates.

## 4. Conclusions

In this work, we showed that it is possible to easily, inexpensively, and rapidly produce well-aligned PLGA nanofibers over remarkably vast areas, achieving uniformly coated surfaces of many cm^2^ and nanofiber lengths in the range of several cm. The directional growth was obtained thanks to an improvement of an already-known and simple technique, called ASB-SANS, which is based on a ternary solution that is deposited onto a given substrate. The ternary solution includes a Sublimating Substance (SS, in this case (−)-menthol) able to crystallize, PLGA, and an Auxiliary Solvent (AS) to keep the system at the liquid state. The here-demonstrated modification of the ASB-SANS method consists of adding a menthol crystal seed to the ternary solution deposited on the substrate immediately after the AS evaporation. The whole system is hence kept at a temperature slightly lower that of the solidification of the Sublimating Substance. Using this procedure, the seed crystal triggers the uniform and directional crystallization of the Sublimating Substance via concurrent epitaxial growth at multiple nucleation sites.

The so-grown aligned PLGA nanofibers showed significant adhesion to different substrates (glass, polyimide, and Si/SiO_x_), and to oxygen-plasma-treated polyimide-coated glass, even when immersed in PBS solution and kept at physiological temperature (37 °C).

Finally, the Directional ASB-SANS protocol showed the ability to produce PLGA nanofibers also on heterogeneous (in terms of presenting both different materials and geometrical discontinuities) substrates, keeping a constant alignment, morphology, and topology.

These results pave the way for practical applications of Directional ASB-SANS in oriented, nanostructured coatings for neural interface systems. Tests of neural cell growth on these substrates have already been started and will be reported soon.

## Figures and Tables

**Table 1:** Compositions of the tested ternary solutions.

Solution ID	Solution Composition		
	AS (mL)	SS (mg)	TM (mg)
M400	0.1	40	0.1
M200	0.1	20	0.1
M100	0.1	10	0.1
M50	0.1	5	0.1

## Data Availability

The data presented in this study are available upon request from the corresponding author.

## Supplementary Materials

The following are available online at https://www.mdpi.com/article/10.3390/ma15020687/s1: Table S1: “Compositions of the tested ternary solutions based on PDCB as Sublimating Substance”; Figure S1: “Topology of PDCB-based ASB-SANS-generated nanostructures”; Figure S2: “FT-IR spectra of PLGA”; Figure S3: “Optical photograph of randomly oriented domains of PLGA-incorporating menthol crystals”; Figure S4: “Optical photograph of the whole system used for the directional ASB-SANS procedure”; Figure S5: “Optical micrographs of PLGA nanofibers grown on glass/PI substrates”; Video SV1: “Formation of radially oriented menthol/PLGA crystals”.
