# Supplementary material for: Directional Growth of cm-Long PLGA Nanofibers by a Simple and Fast Wet-Processing Method
Source: Materials (Basel). 2022 Jan 17;15(2):687. doi: 10.3390/ma15020687 (PMC8777905; doi:10.3390/ma15020687)
Supplement: Supplementary file 1 [file materials-15-00687-s001.zip › materials-1450447-supplementary/materials-1450447 Supplementary Material for publish.pdf]

materials MDPI

Supplementary Materials

Directional Growth of cm-Long PLGA Nanofibers by a Simple and Fast Wet-Processing Method

Erik Betz-Güttner [1], Martina Righi [2], Silvestro Micera [2], and Alessandro Fraleoni-Morgera [2,3,4]*

[1] Department of Physics, University of Trieste, Via A. Valerio, 2, 34127 Trieste, Italy; ERIK.BETZGUTTNER@phd.units.it
[2] Sant'Anna School of Advanced Studies, The Biorobotics Institute, Viale R. Piaggio, 34, 56025, Pontedera (Pisa), Italy; martina_righi@fas.harvard.edu (M.R.); silvestro.micera@santannapisa.it (S.M.)
[3] Department of Engineering and Architecture, University of Trieste, Via A. Valerio, 6/1, 34127 Trieste, Italy
[4] Department of Engineering and Geology, University of Chieti-Pescara, Viale Pindaro 42, 65127, Pescara, Italy
* Correspondence: alessandro.fraleoni@unich.it

Supplementary Materials and Methods

The protocol for ASB-SANS conducted using PDCB as SS is described in detail in reference [13] of main paper.

In Table 1 the compositions of the first tested ASB-SANS ternary solutions, using PDCB as Sublimating Substance, are reported. These solutions have been used to produce the patterns imaged in Figure S1, and the nanofibers growth step has been carried out at room temperature, as described in paragraph 2.3 of the main paper.

Table S1. Compositions of the tested ternary solutions based on PDCB as Sublimating Substance.

Solution ID	Solution composition		
	AS (mL)	SS (mg)	TM (mg)
PDCB400	0.1	40	0.1
PDCB200	0.1	20	0.1
PDCB100	0.1	10	0.1
PDCB50	0.1	5	0.1

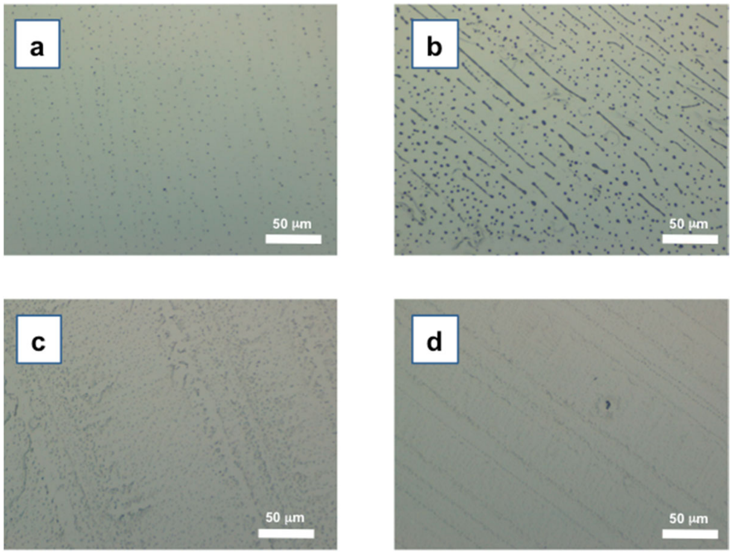

Figure S1. - Topology of PDCB-based ASB-SANS-generated nanostructures, obtained from samples PDCB400 (**a**), PDCB200 (**b**), PDCB100 (**c**), PDCB50 (**d**).

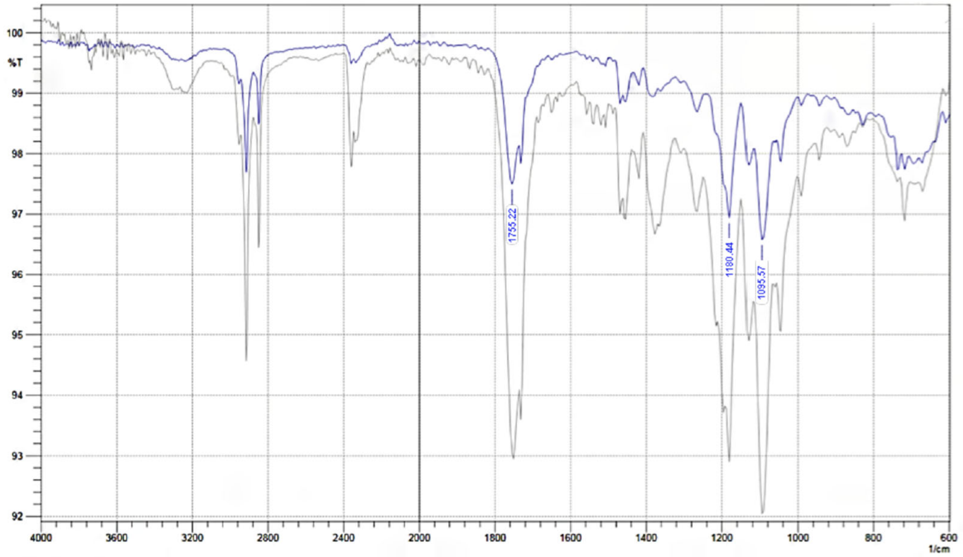

Figure S2. FT-IR spectra of a pure PLGA film from chloroform (brownish curve) and of PLGA nanofibers produced by ASB-SANS using menthol as sublimant (blue curve), showing the absence of noticeable signals signal attributable to compounds different from the menthol itself.

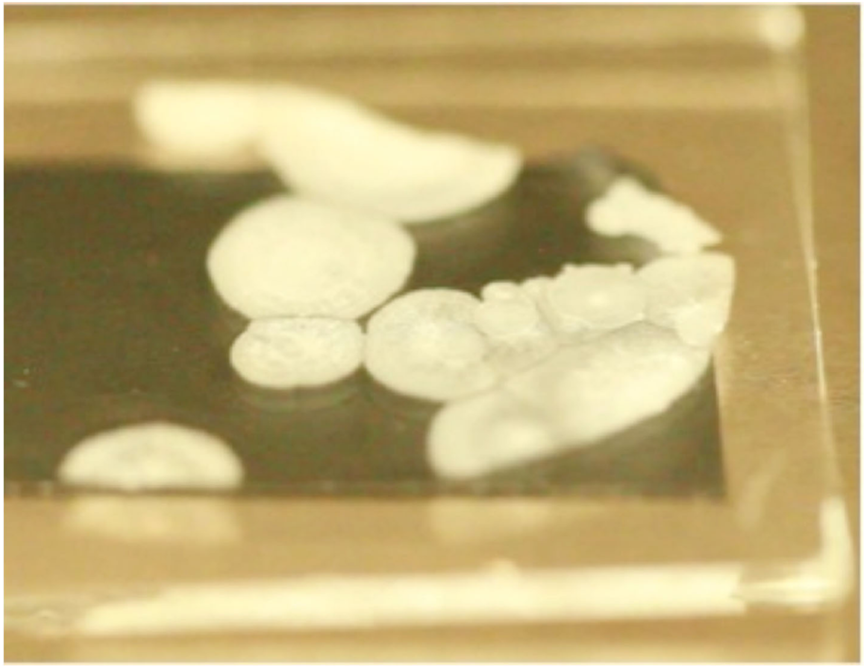

Figure S3. Randomly oriented domains of PLGA-incorporating menthol crystals obtained by non-directional, thermally controlled ASB-SANS procedure.

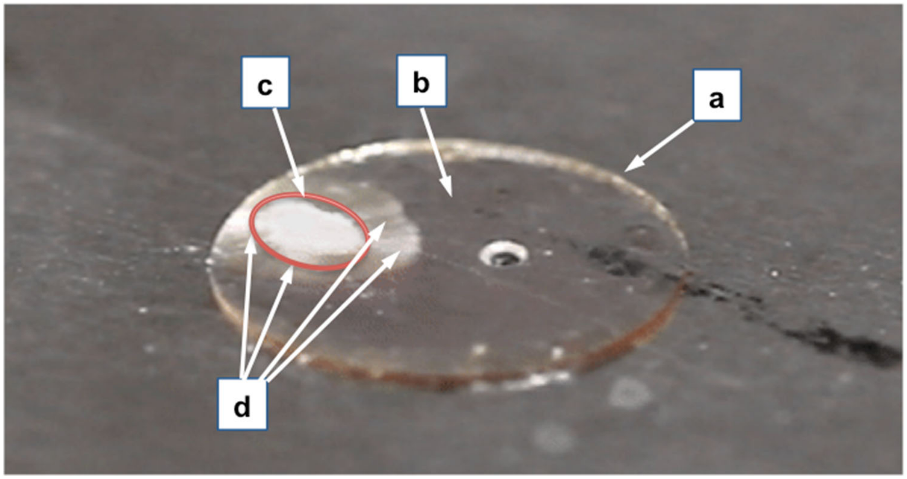

Figure S4. Photograph of the whole system used for the directional ASB-SANS procedure: polyimide-coated substrate (**a**), ASB-SANS ternary solution constituted by menthol, chloroform and PLGA (**b**), menthol single crystal (inside the red ellypsis) used as seed for promoting uniform growth of the menthol/PLGA layer (**c**), oriented polycrystals forming in the ternary solution due to the multi-epitaxial effect of the seed menthol crystal (**d**).

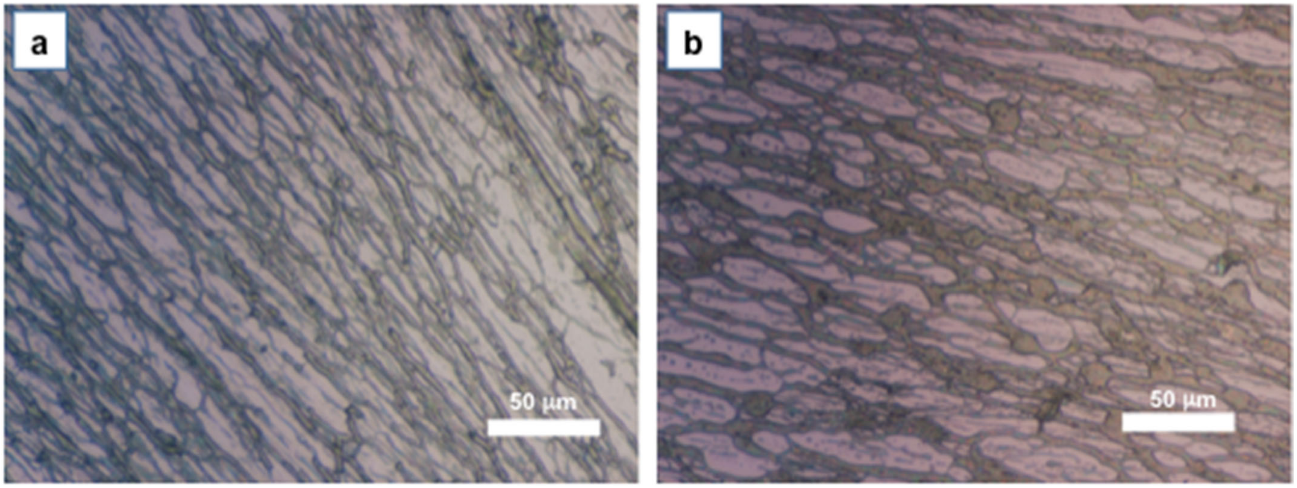

Figure S5. Optical micrograph of PLGA nanofibers grown on (**a**) glass and (**b**) PI substrates, evidencing slight structural modifications (fibers lateral size enlargement) after 96 hours (4 days) of immersion in PBS at 37 °C.
